# A Scoping Review of Observational Studies on Food and Beverage Advertising on Social Media: A Public Health Perspective

**DOI:** 10.3390/ijerph20043615

**Published:** 2023-02-17

**Authors:** Juliana de Paula Matos, Michele Bittencourt Rodrigues, Camila Kümmel Duarte, Paula Martins Horta

**Affiliations:** Department of Nutrition, Federal University of Minas Gerais, Belo Horizonte 30130-100, MG, Brazil

**Keywords:** food advertising, digital technology, social media, ultra-processed foods, scoping review

## Abstract

Popular social media platforms have been actively used by ultra-processed food companies to promote their products. Being exposed to this type of advertising increases the consumption of unhealthy foods and the risk of developing obesity and other non-communicable diseases (NCDs). Thus, monitoring commercial content on social media is a core public health practice. We aimed to characterize the methods used for monitoring food advertising on social media and summarize the investigated advertising strategies via a scoping review of observational studies. This study is reported according to the MOOSE Statement, and its protocol was registered with the PROSPERO International Prospective Register of Systematic Reviews (registration nº. CRD42020187740). Out of the 6093 citations retrieved, 26 met our eligibility criteria. The studies were published from 2014 to 2021, mostly after 2018. They focused on Australia, Facebook, strategies aimed at children and adolescents, and advertising practices of ultra-processed food companies. We grouped strategies in eight classes: post features (n = 18); connectivity and engagement (n = 18); economic advantages, gifts, or competitions (n = 14); claims (n = 14); promotional characters (n = 12); brand in evidence (n = 8); corporate social responsibility or philanthropy (n = 7); and COVID-19 (n = 3). We found similarities in the investigation of strategies regardless of the type of social media. Our findings can contribute to the designing of tools for monitoring studies and regulatory mechanisms to restrict the exposure of food advertising.

## 1. Introduction

Food marketing comprises advertising activities designed to promote the consumption of food or beverages, such as advertisements on traditional media (television, radio, press media, etc.) and the new internet media (websites, social media, applications, etc.) [1]. Modern mechanisms of exposure are used for an advertisement to reach a high number of individuals, while mechanisms of power that involve investments in design, creative content, and execution guarantee the transmission of messages with highly persuasive content [2]. Strategies commonly used in food advertising include celebrity/sports endorsements, promotional characters, gifts/incentives, tie-ins, branding, persuasive appeals (e.g., fun, taste), health/nutrition claims, and disclaimers, among others [3]. As a result, food advertising exposure influences the way individuals perceive, desire, choose, and consume food and helps increase the demand for an advertised product [2,4,5,6,7]. The impact of food advertising exposure is greater among children because they possess limited cognitive abilities to differentiate an informative tone from commercial content [3].

Food advertising is focused on ultra-processed food (i.e., products that are rich in saturated fats, sodium, added sugars, and limited in protective nutrients) [8,9,10]. This is concerning since the consumption of these food products is linked with obesity and other non-communicable diseases (NCDs) [11,12]. A systematic review and meta-analysis of 43 observational studies showed a consistent relationship between the consumption of ultra-processed foods and overweight, obesity, metabolic syndrome, cardiometabolic diseases, and all-cause mortality [11]. In an experimental study, Hall et al. (2019) concluded that adults exposed to ultra-processed diets consume more energy and gain more body weight than individuals exposed to an unprocessed diet [12]. Contextual factors that stimulate individuals to consume ultra-processed food are of great concern given the global increase noted in the sales of ultra-processed food in the last two decades [13,14].

Recently, with the growing advancement of digital technologies and online communication, food advertising has penetrated the digital environment [15]. On this medium, food advertising is mainly presented on social media platforms (e.g., Facebook, Instagram, Twitter, and YouTube) where food companies can connect directly to users/consumers and use engagement techniques and behavioral data tracking to direct the advertising message, which is known as programmatic media [16]. Social media is a technology-centric ecosystem in which a diverse and complex set of behaviors, interactions, and exchanges involving various kinds of interconnected actors (individuals, firms, organizations, and institutions) can occur. Social media is pervasive, widely used, and culturally relevant [17]. In 2021, 4.5-billion social media users were estimated worldwide [18]. In addition, Rummo et al. estimated that 6.2-million adolescents followed the Twitter and Instagram accounts of 27 of the most highly advertised fast food, snack, and drink brands in 2019 in the world [19]. The number of adolescents following the selected brands’ accounts is higher than the number of users following any other account on Twitter [19].

Some studies have monitored food advertising on social media platforms and examined: (i) the advertising strategies most used by food companies to communicate in the digital environment; (ii) the target audience for advertising messages; and (iii) the main social media platforms used. However, apparently, there is great heterogeneity in the methods of measuring food advertising, and there seems to be a concentration of studies in a few countries [20,21].

Some tools have also been proposed to monitor food advertising. In 2018, the World Health Organization Regional Office for Europe (WHO/Europe) launched CLICK, an online marketing monitoring tool for unhealthy foods aimed at children and adolescents. Among its objectives, CLICK seeks to comprehend the digital ecosystem and investigate real exposure to food marketing [22]. As a way of standardizing the application steps of this tool, in 2020, WHO/Europe launched specific protocols for collecting power marketing strategies on social media pages, websites of the food industry, and digital influencers’ pages (i.e., content creators who use their pages or channels on social media platforms to influence consumers’ behavior and purchasing decisions to benefit brands or products) [20,23]. Both initiatives aim to generate replicable and comparable results across various digital media platforms in different countries. However, at the moment, no study has applied all recommendations present in these protocols, and until now, no specific recommendations are focusing on young people or adults.

Monitoring food advertising is a core public health practice to better understand the individuals’ exposure to the commercial content of unhealthy food and to address public health policies aiming to limit this exposure [20]. A systematic review that summarized the influence of unhealthy food and beverage marketing through social media and advergaming on diet-related outcomes in children suggested this type of marketing has a significant effect on pester behaviors, as well as the food choices and intake of children [24]. Among adolescents, the evidence also shows these individuals are more likely to share unhealthy food posts and recall and recognize a greater number of unhealthy food brands when exposed to this content. They also rate peers more positively when they had unhealthy posts in their feeds [25]. Additionally, constant exposure to unhealthy food advertisements on social media can impair young adults’ ability to adopt healthy eating behaviors, causing feelings of guilt [26]. A systematic review assessed the relationship between digital marketing and young people’s (12 to 30 years) attitudes and behaviors towards unhealthy commodities. One of the key findings was that marketers used peer-to-peer transmission of messages on social networking sites (e.g., friends’ likes and comments on Facebook) to blur the boundary between marketing contents and online peer activities [27]. Despite this, currently, few restrictions on food advertising are applicable to social media [28]. Thus, summarizing the advertising strategies used by the food industry to advertise their products on social media can guide the design of new regulations on this topic.

Therefore, our objectives are:

(i) To characterize the methods used for monitoring food advertising on social media.
(ii) To identify and summarize the investigated advertising strategies, according to different social media platforms and target audiences.

Our results will help understand the nature of what has been investigated, the weaknesses, and the methodological potential of the research, and they will provide information that can be used to design tools for monitoring food advertising. Consequently, this study contributes to the efforts made to control and reduce obesity and NCD occurrence worldwide.

## 2. Materials and Methods

This is a scoping review of observational studies investigating food advertising on social media. This type of review is recommended in cases when the researchers aim to analyze the current state of research on a particular topic or field and to identify and examine knowledge gaps [29]. Thus, a scoping review is deemed the most appropriate method to achieve our research objectives, which are to map the methods used to identify food advertising strategies in social media and summarize them.

This study is reported according to the MOOSE Statement [30], and its protocol was registered with the PROSPERO International Prospective Register of Systematic Reviews (registration nº. CRD42020187740).

### 2.1. Search Strategy

The search was carried out in the databases EMBASE, MEDLINE, Web of Science, and Scopus. Furthermore, the reference lists of included publications were revised. Language and time restrictions were not applied to the search strategy. The searches in the databases were conducted in May 2021. The search strategy was based on the SPIDER framework [31]. Based on this tool, we defined: the Sample—S (“Social media”), Phenomenon of Interest—PI (“Methodologies used to evaluate food advertising”), Design—D (“Advertising”), Evaluation—E (“Food advertising”), and Study Type—R (“Observational studies”). The search terms were based on the literature [19,20] and the definition of broad terms in the theme. In both situations, the terms were consulted in the Medical Subject Headings (MeSH terms), and new related terms were also included.

The following terms and derivatives were used to perform the searches: social media, online, Facebook, Instagram, Twitter, YouTube, food, fast food, food and beverages, beverages, carbonated beverage, artificially sweetened beverages, sugar-sweetened beverages, food industry, meals, snacks, food brands, food companies, Coca-Cola, PepsiCo, Mondelez, Unilever, Nestlé, Danone, chips, McDonald’s, Burger King, Subway, Kentucky Fried Chicken, Starbucks, ultra-processed, industrialized foods, food marketing, advertising, propaganda, advertisement, communication, persuasive communication, information dissemination, digital marketing, digital media, marketing techniques, marketing strategies, marketing techniques, trends marketing, vehicles of marketing, publicity, persuasive marketing techniques, junk food marketing, sports sponsorship food, food sponsorships, fast food marketing, unhealthy food marketing, unhealthy food and beverages marketing, food and beverages marketing, food advertisements, marketing of food, influencer marketing of food, food publicity, food promotion, persuasive food marketing, monitoring food marketing, power food marketing, and online food communication. The search strategies used on each database are available in Supplementary Material S1.

### 2.2. Eligibility Criteria

The criterion for inclusion was restricted to observational studies that evaluated food advertising strategies on social media (e.g., Facebook, Instagram, YouTube, and Twitter). We defined food advertising as promotional advertisements for food or beverages (except alcoholic beverages) on different social media platforms containing a variety of advertising strategies. The exclusion criteria included the following: (i) studies not published in a scientific journal; (ii) studies without enough information available to define eligibility or unsuccessful contact with the researcher; (iii) studies without an advertising strategy assessment (i.e., studies that did not identify the advertising strategies used to promote food brands and food product); (iv) studies without an assessment on social media, such as those that evaluated food advertising on television, print media, food stores, outdoor billboards, and food labelling; (v) studies identifying the exclusive advertising strategy of breast milk substitutes; (vi) studies that measured individuals’ exposure to food advertising on social media; (vii) studies only including cigarette or alcohol advertising; and (viii) any duplicate or redundant publications. The publication language was not an eligibility criterion.

### 2.3. Study Selection, Data Extraction, and Analysis

Titles and abstracts of the selected references were double-checked by two investigators (JdPM and MBR) for eligibility criteria, with differences solved by consensus. We carried out a manual search through all reference lists of selected studies to include studies not identified in the search strategy. However, after this search, we did not add any studies. We used the software ENDNOTE X9 and ENDNOTE 20 to read the titles and abstracts. Full versions of the selected abstracts were also double-checked by the same two investigators. Included studies had their data extracted independently and in duplicate by two investigators (JdPM and MBR). The extracted data were: (i) the authors and year of publication; (ii) coverage, such as country or countries; (iii) social media platform; (iv) target audience (children, adolescents, young adults, or general); (v) the unit of analysis (pages/channels or posts/videos/tweets); (vi) type segment of food or beverages brands (vii); sample definition criteria; (viii) time range for data collection; (ix) type of content analysis (methodology used to obtain the advertising strategies; can be qualitative or quantitative).

The advertising strategies identified were extracted from each publication, and the number of publications using each advertising strategy was counted. To identify and summarize the advertising strategies, an inventory was carried out. From an initial list containing all the advertising strategies extracted from the studies, two investigators (JdPM and MBR) gathered similar strategies (for example, “Celebrities” and “Sportspeople”), based on the definition specified by the authors in each study, and organized them into groups with similar themes (i.e., “Promotional characters”). This step was carried out considering a denomination that enclosed all of the themes. In some cases, we were inspired by groups of strategies previously found in assessed studies or food marketing data collection protocols on television [32], the internet [23], or multiple media [3]. The grouping was discussed with a third researcher (PMH) and adjustments were made.

Since the nature of monitoring studies of food advertising strategies on social media varies across social media platforms and target audiences, all identified groups of advertising strategies were stratified by these variables, in addition to being described in the general sample. Social media platforms were grouped based on the typology proposed by Zhu and Chen [33], which divides social media according to the nature of the connection and the level of customization of the messages. Based on this reference, social media platforms’ purposes were characterized as relationship (Facebook), self-media (Twitter), and creative outlet (Instagram or YouTube) [33].

### 2.4. Risk for Bias Assessment

The Newcastle–Ottawa Scale [34] is a star system scale used to assess the quality of cohort studies, which was adapted for the purposes of this scoping review, excluding assessments about comparability and follow-up. The scale presents two main sections: Part I, which is the selection of the study (a maximum of five stars); and Part II, which is the assessment of the outcome (a maximum of two stars).

Part I includes four specific items to be assessed: (i) sample representativeness (according to the aim of the study and the number of media and brands studied); (ii) sample analysis period (whether data collection was assessed in an acceptable length of time according to the objectives of the study); (iii) identification of the exposure (refers to the tools used to identify the strategy); and (iv) definition of the exposure (refers to the detailed definition of the strategy studied).

Part II includes the assessment of the outcome. This refers to the methodology used by the research team to analyze data on advertising strategies. In this section, we removed items that assessed the quality of statistical analyses because this criterion did not conform to the analyzed studies’ profile (most were descriptive studies).

Two authors evaluated the risk of bias. Differences in quality assessment scores between investigators were unusual and solved by consensus. When a consensus could not be reached, a third investigator evaluated the scores. The adapted scale is available in Supplementary Material S2.

## 3. Results

### 3.1. Study Selection

**Figure 1:**
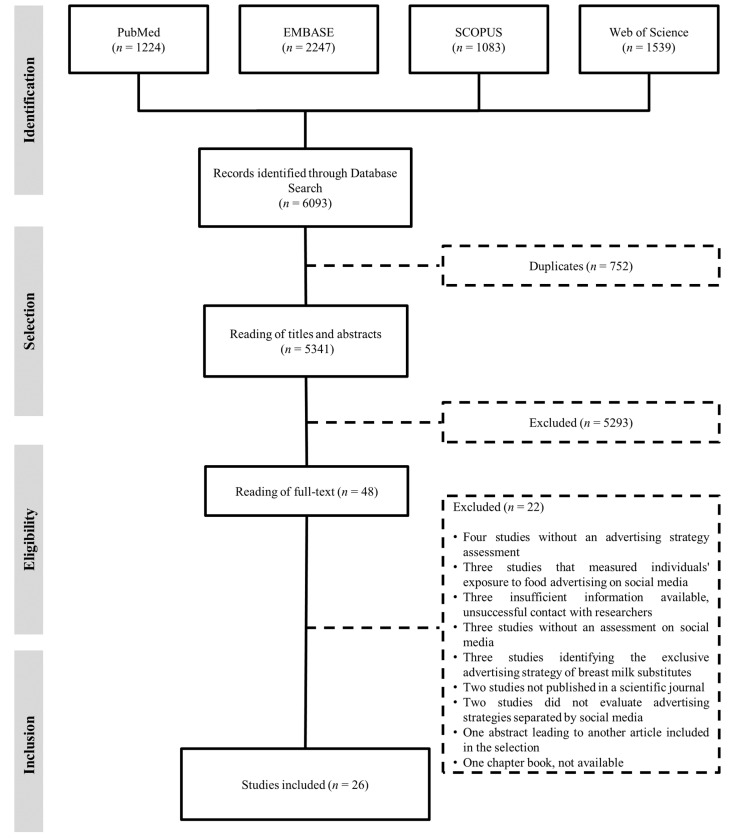
Flowchart showing the process of article selection (May 2021).

Searches in the databases identified 6093 citations, with 752 duplicates that were removed, resulting in 5341 records that were selected for the evaluation of the title and abstract. Forty-eight abstracts were selected for the full-text assessment. A total of 26 articles met the eligibility criteria and were selected for the scoping review [35,36,37,38,39,40,41,42,43,44,45,46,47,48,49,50,51,52,53,54,55,56,57,58,59,60]. The studies were found only in English (n = 25) and Spanish (n = 1). The selection process is summarized in Figure 1.

### 3.2. Study Characteristics

The characteristics of the selected studies are presented in Table 1.

Studies were published between 2014 and 2021, with 2018 being the year with the largest number of publications (n = 9) [41,42,43,44,45,46,47,48,49].

Most of the studies were carried out in Australia (n = 10) [35,36,37,43,45,46,47,48,51,59] and Spain [54,55]. Single studies were carried out in Brazil [42], Uruguay [58], Malaysia [44], Peru [41], Thailand [50], and the United States [53].

Nine studies investigated food advertising on more than one social media platform [43,46,48,49,51,53,56,57,59]. Individually, Facebook was the most evaluated media (n = 9) [35,36,37,41,42,47,50,52,58] in contrast to Instagram (n = 2) [40,45], YouTube (n = 3) [44,54,55], and Twitter (n = 3) [38,39,60]. In a temporal analysis, we observed that Facebook, Twitter, Instagram, and YouTube have been studied since 2014, 2016, 2017, and 2018, respectively. From these years, all platforms continued to be evaluated continuously.

A larger portion of the studies focused on food advertising aimed at specific audiences, such as children and adolescents (n = 10) [36,37,40,41,44,49,50,53,54,55]. The other studies (n = 16) focused on food advertising aimed at young adults (n = 4) [43,45,47,48] or the general public (n = 12) [35,38,39,42,46,51,52,56,57,58,59,60]. The definition for each age group is unique and varied between studies.

Only two studies evaluated child-centric YouTube channels [54,55], while other investigated contexts were a university’s social media (n = 1) [43] and family-friendly event pages (n = 1) [57]. Among the studies, 22 informed the volume of posts/videos/tweets studied. The majority (n = 12) evaluated up to 500, seven evaluated over 500 up to 1000, and only three evaluated more than 1000. Overall, this volume of posts/videos/tweets ranged from 21 to 3672, and from 6 to 13 for pages/channels. Additionally, a great variation was observed among the studies on the time interval adopted for data collection. Studies with the shortest period of data collection ranged from one to three weeks [40,44], and two studies investigated food advertising for three or more years [35,60]. The majority of the sample (n = 22) collected data for a period between one and twelve months [36,37,38,39,41,42,43,45,46,47,48,49,50,51,52,53,54,55,56,57,58,59].

Most studies (n = 14) used quantitative content analysis to investigate advertising strategies [35,36,37,40,41,42,43,44,45,49,50,51,52,56], 10 used qualitative content analysis [38,39,46,53,54,55,57,58,59,60], and two used both approaches simultaneously [47,48].

The type of brands and the definition criteria used by the studies to select their samples are presented on Table 2.

The most frequent unit of analysis was official accounts (i.e., commercial pages created by the brands to promote their products and interact with users on social media platforms) of food and beverage brands (n = 24) [35,36,37,38,39,40,41,42,44,45,46,47,48,49,50,51,52,53,54,55,56,58,59,60]. Among them, three investigated only a single brand [38,39,40], while the others ranged from 3 to 96 food and beverage brands [35,36,37,41,42,43,44,45,46,47,48,49,50,51,52,53,54,55,56,57,58,59,60]. Among these studies, the evaluation of unhealthy food and beverage (such as candies, energy drinks, soft drinks, and salty snacks) brands predominated, in addition to fast-food establishments. Examples of evaluated brands are KitKat, Gatorade, Coca-Cola, Lays, and McDonald’s. Brands were usually selected based on their presence in the market, or their popularity on social media or between specific target groups.

### 3.3. Advertising Strategies

Of the 26 studies assessed in this scoping review, in two studies, we could not identify from which social media platform the authors identified advertising strategies [36,52]. This happened because the studies evaluated different platforms simultaneously and did not present separate results for each one. In the remaining 24 studies, eight groups of advertising strategies were extracted and are described in Table 3.

The participation of the eight groups of advertising strategies in the sample is presented in Table 4: post features (n = 18); connectivity and engagement (n = 18); economic advantages, gifts, or competitions (n = 14); claims (n = 14); promotional characters (n = 12); brand in evidence (n = 8); corporate social responsibility (CRS) or philanthropy (n = 7); and COVID-19 (n = 3). This participation is also presented stratified by social media and the target audience.

In the general sample, some advertising strategies stood out among the groups. For example, the most used strategy in the brand by evidence group was branding elements. Strategies such as audiovisual and graphics resources (n = 14), presence of products/food/beverages (n = 8), and post origin (n = 7) were more frequently investigated in the post features group. In the connectivity and engagement group, media tools (n = 11) were most investigated. While in the promotional characters group, celebrities (n = 8), sportspeople (n = 7), and events (n = 7) were the most investigated. Among the claims group, the strategies, feelings, and emotions (n = 10) and product characteristics (n = 8) were frequent in the studies. Finally, encouraging messages (n = 3), home delivery and takeaway (n = 3), CSR or philanthropy (n = 3), isolation activities (n = 3), and maintaining an essential supply chain and the commitment of the brand (n = 3) were the most investigated advertising strategies in the COVID-19 group.

### 3.4. Advertising Strategies by Social Media

Studies that evaluated food advertising on Facebook (n = 7) more frequently investigated the groups of strategies economic advantages, gifts, or competitions (n = 6) and connectivity and engagement (n = 6). For this last group, media tools (n = 4) and conversations (n = 4) were the most investigated strategies. Post features were also frequent on this platform (n = 5), especially for the audiovisual and graphics resources strategy (n = 5).

On Twitter (n = 3), the groups of strategies more frequently investigated were post features (n = 2), connectivity and engagement (n = 2), and claims (n = 2). In the post features group, audiovisual and graphics resources (n = 2) was the most used strategy. In the connectivity and engagement group, the most prevalent strategies were incentive for an action online (n = 1) and conversations (n = 1). Among the claim strategies group, appeals on feelings and emotions were the most common strategy (n = 2).

The studies that assessed Instagram or YouTube (n = 5) investigated advertising strategies from the post features group (n = 4), mainly, audiovisual and graphics resources (n = 3) and post origin (n = 3) strategies. Claims groups were also investigated in four of five studies for these two platforms, especially strategies related to product characteristics (n = 4) and feelings and emotions (n = 3).

For studies that monitored multiple media (n = 9), the most investigated strategies groups were post features (n = 7), connectivity and engagement (n = 7), and claims (n = 6). These strategies had as main representatives: presence of products/food/beverages (n = 5 as for post features), media tools (n = 5 as for connectivity and engagement strategies), encouragement to action (n = 3), and feelings and emotions (n = 3) as for claims.

### 3.5. Advertising Strategies by Targeted Audience

Studies focusing on the evaluation of advertising strategies targeted at children or/and adolescents (n = 9) had post features (n = 6), claims (n = 6), economic advantages, gifts, or competitions (n = 6), and connectivity and engagement (n = 6) as the most frequently investigated advertising strategies. The most notable strategies targeted at this public were: audiovisual and graphics resources (n = 4) in the post features group; product characteristics (n = 5) in the claims group; and media tools (n = 5) in the connectivity and engagement group.

All studies focusing on young adults (n = 4) investigated in the post features strategies groups (n = 4) - with an emphasis on the audiovisual and graphics resources strategy (n = 3) - and in the connectivity and engagement strategies group (n = 4) - with an emphasis on media tools (n = 2) and conversations (n = 2).

Finally, studies focusing on the general public (n = 11) frequently investigated groups of strategies such as post features (n = 8) and connectivity and engagement (n = 8). In the post feature strategies group, the investigation of audiovisual and graphics resources (n = 7) was common. In the connectivity and engagement strategies group, the incentive for an action (online) (n = 5) and conversations (n = 5) strategies were the most investigated.

### 3.6. Risk for Bias

Nine studies presented a maximum score (seven stars) on quality assessment, and 12 presented six or five stars. Five studies presented scores equal to or less than four stars. The parameter that most influenced the quality assessment of the studies was the short period of sample analysis; 11 of the 25 studies did not score for this criterion. The assessment of risk for bias is presented in Table 1, with details presented in Supplementary Material S3.

## 4. Discussion

This scoping review summarizes the methodologies proposed by observational studies for monitoring food advertising on social media. There was a large concentration of studies published from 2018 and carried out in Australia. The majority investigated official social media pages of unhealthy food brands, mainly on Facebook. The investigated marketing strategies were very similar across social media platforms, although some particularities were noted. Studies that monitored advertising aimed at children or/and adolescents were predominant and employed a variety of advertising strategies. Our results demonstrate that observational studies aiming at monitoring food advertising on social media are still incipient and do not capture the actual exposure of individuals to the commercial content, despite the high growth and popularization of these platforms.

The high concentration of studies in Australia reveals the country’s leading role in food advertising research. According to a systematic scoping review, Australia is among the top five countries that has most published about the digital food environment, including digital food marketing [21]. In addition, an Australian study was the first to describe the advertising content present on food brands’ pages on Facebook [35]. This study was cited by 26.9% of this review sample as a reference for defining data collection procedures [41,42,43,45,47,48,50]. Therefore, expanding food advertising monitoring to other countries, including those with low- and medium-income levels, is important to advance the understanding of food advertising exposure worldwide.

Most studies analyzed advertisements carried out by unhealthy food and beverage brands and fast-food establishments on their official pages. However, exposure to digital advertising is not only limited to this content; it also occurs through programmatic media. However, no study included in our sample has measured this type of advertising. Therefore, going beyond monitoring only the advertising content generated by food brands on their official pages and social media is necessary to draw a wider picture of the real exposure of individuals to food advertising.

Moreover, commercial content produced by digital influencers can also persuade individuals. In our review, these studies comprised only 7.7% of the sample (n = 2) [54,55]; more studies are necessary in this field owing to its growth in recent years [61]. Food brands invest in digital influencers because they reach a great number of individuals and also construct a relationship of trust with their followers, especially children and adolescents [5]. For example, children who viewed featured images of influencers with unhealthy snacks on Instagram had significantly increased overall calorie intake and the intake of unhealthy snacks, compared with children who viewed influencers with non-food products [5].

According to another finding, food advertising monitoring studies focus on Facebook. This platform was launched in 2004, while YouTube, Twitter, and Instagram emerged later, in 2005, 2006, and 2010, respectively. In terms of popularity, Facebook is still the most accessed social media in the world (with 2895-billion users) [62] and the most used by marketers [63]. However, other social media platforms are almost as popular. YouTube has 2291-billion users, WhatsApp has 2000 billion, Instagram has 1393 billion, Weixin/WeChat has 1251 billion, TikTok has 1 billion, Snapchat has 557 million [62], and Twitch has 7.57 million [64]. Some of these platforms are more popular among young adults and teens, and they should also be studied with regard to the presence of food advertising. Although two studies have shown a wide exposure to unhealthy food marketing on Twitch, a popular platform where individuals broadcast live audiovisual content, these studies did not evidence the power strategies used to promote these foods [65,66].

In terms of the marketing strategies present on social media, regardless of the type of platform, we identified the high investigation of (i) the audiovisual and communication resources almost always exclusive to social media, such as photos, videos, links, hashtags, etc.; (ii) the offer of economic benefits; (iii) people (celebrities and sportspeople) or elements endorsing the commercial message, such as cartoons/company-owned characters or licensed characters, sponsorships and partnerships, among others; (iv) appeals that emphasize product characteristics; (v) strategies that reinforce the brand identity, such as logos, colors, trademarks, or slogans; and (vi) the strategies of CSR or philanthropy, mainly during the COVID-19 pandemic. Except for the first and last group of advertising strategies, all the others are usually investigated when monitoring food marketing on television [67]. This may be a consequence of using the same data collection methods on social media that were originally designed for monitoring food advertising on television.

Despite the similarities among social media discussed above, some differences in the investigation of advertising strategies were identified among the platforms. The offer of economic advantages, gifts, or competitions was more common in studies on Facebook than in studies that evaluated advertising on Twitter, YouTube, or Instagram. On the contrary, the claims strategy was more frequent in studies that evaluated these three platforms in comparison to Facebook, as product characteristics claims were more prevalent on YouTube or Instagram. Additionally, studies that evaluated advertising on Twitter and Facebook more commonly investigated connectivity and engagement strategies, with media tools predominant on Facebook, while the incentive for an action (online) strategy appeared only in studies that investigated Twitter.

The great homogeneity among the studies regarding the investigation of some advertising strategies was considered a point for improvement. For Zhu and Chen [33], platforms like Facebook are used to build and maintain connections between people through the exchange of messages between close people or friends. Twitter is characterized as a self-media platform in which people connect to profile-based accounts that are established by well-known organizations, celebrities, or products. YouTube or Instagram, on the other hand, includes services that facilitate individuals’ sharing of interests, creativity, and hobbies with other people of similar interests [33]. As each platform offers different experiences to users and a unique context for advertising, we point out that monitoring studies should investigate different advertising strategies according to each social media platform typology.

As for the target audience of the studies, we observed a large variety of advertising strategies employed when a study monitored advertising aimed at children and adolescents. On the other hand, for studies focusing on the young adults or the general public, the emphasis was only on the investigation of post features and connectivity and engagement strategies.

In line with our results, a rapid review showed the most frequent strategies used to direct the commercial message to children on television, internet, packaging, print materials, and multiple platforms were those involving connectivity dimensions such as games, advergames or other interactive activities, and free downloads for children (coloring sheets, wallpapers/screensavers, comics, e-books, etc.). Economic benefits (giveaways to be redeemed later, such as contests, competitions, coupons, special order items, and product samples) and claims (nutrition-related claims such as nutrient content and ingredient information) were also frequent [10].

The vulnerability of individuals up to 12 years of age to the advertising content explains the focus on studying advertising strategies aimed at persuading children and adolescents [68]. However, young adults can also be vulnerable to the commercial content. A sample of 166 Australians, ranging from 18 to 24 years old, exposed to unhealthy food advertisements reported difficulty realizing healthy eating behaviors and developed feelings of guilt [26]. Furthermore, a review showed a significant positive association between food advertising and food choices among adults [69]. Although adults have a greater understanding of the persuasive intent of advertising, this does not make them exempt from being influenced and less vulnerable to its impacts. An important step to advance in the monitoring of food advertising is to comprehend the strategies used by companies to communicate with the adult audience. After that, monitoring protocols must be updated to address these strategies.

Finally, we highlight that it is still a challenge to successfully distinguish between advertisements and non-commercial publication content. Advertisements involve hybridity between brand content and entertainment, such as unboxing, product placement, and advertising via influencers [20,61]. This makes the identification of the commercial content a difficult task, and this hybridity is predominant on social media. More transparency when reporting advertising action by advertisers (e.g., the food industry, platforms, and third parties) is necessary. Additionally, researchers in this field must extend their backgrounds beyond public health. They need to invest in knowledge of the tools and strategies in the field of marketing and communication to learn about the trends of commercial communication in the digital environment. New approaches in collecting and monitoring data should also be adopted by researchers. These need to consider any food display in social media content, even if it does not indicate an advertising action (e.g., displaying food or brands in the background or just in speech, without the direct illustration).

Overcoming all the aforementioned difficulties faced in monitoring food advertising on social media can orientate other investigations that aim to describe characteristics of the food advertising content to which individuals are being exposed and, consequently, can help improve current regulations seeking to protect individuals from the persuasive content that promotes unhealthy foods in the online environment. Therefore, this review contributes to this field by adding information on the methods used to monitor food advertising on this medium and the constraints to be surpassed.

### 4.1. Policy and Social Implications

The results of this scoping review are useful for public health purposes. Guidelines aiming to protect individuals from food advertising content can restrict certain strategies that are most used by ultra-processed food brands to persuade consumers. For example, in Portugal, the Law No. 30/2019 prohibits unhealthy food brands to use some advertising strategies aiming at minors under 16 years of age, including in social media. One of the restrictions applies to messages that stimulate the exaggerated consumption of the advertised foods [70]. Based on our results, many of the strategies that were identified could be included in the list of restricted strategies in this regulation and in others. The list of restrictions can be defined considering differences between age groups and social media platforms.

In addition, our results also can be useful in promoting programs focused on media literacy among different audiences. In the digital environment, marketing media literacy aims to encourage individuals to develop the ability to use knowledge and skills to understand the purpose of advertisements that promote brands and products through media platforms, mobile apps, and electronic devices [71]. The evidence has shown that the first action to achieve marketing media literacy is to be aware of the marketing formats and media used [72].

### 4.2. Limitations

Some limitations were faced when running the study. First, we did not estimate the frequency of each group of advertising strategies in the sample because a high heterogeneity was found in the way each study described its results. This approach could have enabled the summary of the profile of food advertising on social media. Second, by our eligibility criteria, studies that had evaluated the impact of food advertising exposure on individuals’ behaviors were not included in our sample, as the results of the experimental design require a particular interpretation. However, they could have also investigated advertising strategies, although we did not expect different approaches from those described in this review. Third, we highlight, despite not being an exclusive limitation of the present study, that due to the absence of studies carried out on all social media platforms currently available (e.g., Tik Tok), it does not allow us to extrapolate the results evidenced for all of them. Despite these limitations and challenges in monitoring food advertising, we highlight that most of the studies presented adequate methodological quality. Finally, given the recent research on the subject and centrality in a few countries, a limited number of studies were available. However, in the following years, a greater number of investigations is expected on food marketing monitoring from a public health perspective.

## 5. Conclusions

Observational studies that monitored food advertising on social media are recent, not widely disseminated across countries, centered on one social media platform—Facebook— and focused on food advertising aimed at specific audiences, especially children and adolescents. They evaluate different pages of brands of unhealthy foods in the countries.

As for the identification of advertising strategies, we evidenced/carried out a varied investigation, covering eight different groups. These strategies were similar between different social media platforms, with a greater diversity of use among studies that monitored advertising aimed at children and adolescents.

Future research can focus on (i) monitoring food advertising on social media in countries with low- and medium-income levels and in prominent platforms; (ii) going beyond monitoring only the advertising content generated by food brands on their official pages; (iii) investigating advertising strategies according to the purpose of each social media, instead of using the same protocol regardless of the type of platform; (iv) monitoring advertising content directed to adults and the elderly and studying how these groups are affected by this type of message; and (v) proposing methods to facilitate the identification of the commercial content of different types of messages spread on social media. The results from these studies, in addition to those provided by the current study, will help researchers to design tools for monitoring food advertising on social media.

## Figures and Tables

**Table 1:** Characteristics of the selected studies.

Authors	Coverage	Social Media ^2^	Target Audience ^3^	Unit of Analysis	Time Range	Type of Content Analysis	Risk for Bias ^4^
[35]	Australia	Facebook	General	27 brands’ pages	From the page creation to February 2013 (mean = 3.65 years per page)	QT	5
[36]	Australia	Facebook	Children (≤12 y) and adolescents (13–17 y)	21 advertisements from 3 brands’ pages	2 months (1 June to 31 July 2013)	QT	6
[37]	Australia	Facebook	Children or/and adolescents	6 brands’ pages	6 months	QT	6
[38]	Global	Twitter	General	200 tweets from 1 brand’s page	20 months (October 2012 to June 2014)	QL	7
[39]	Global	Twitter	General	565 tweets from 1 brand’s page	12 months (2014)	QL	7
[40]	Unreported	Instagram	Children or/and adolescents	128 posts from 1 brand’s page	1 week (24 April 2015 to 1 May 2015)	QT	5
[41]	Peru	Facebook	Children and adolescents (6–16 y)	515 posts from 48 brands’ pages	1 month (August 2016)	QT	6
[42]	Brazil	Facebook	General	16 brands’ pages	12 months (September 2014 to October 2015)	QT	7
[43]	Australia	Twitter, Facebook, and Instagram	Young adults	417 posts published on a university’s page	12 months	QT	4
[44]	Malaysia	YouTube	Children or/and adolescents	250 videos from 25 YouTube channels	3 weeks (October 2017)	QT	6
[45]	Australia	Instagram	Young adults	3672 posts from 15 brands’ pages	12 months (15 March 2015 to 15 March 2016)	QT	7
[46]	Australia	Facebook and Instagram	General	79 posts from 17 brands’ pages	2 months (August and September 2017)	QT	6
[47]	Australia	Facebook	Young adults (13–25 y)	446 posts from 6 brands’ pages	6 months (1 January 2015 to 30 June 2015)	QL/QT	4
[48]	Australia	Facebook, Twitter, and YouTube	Young adults	624 posts from 9 brands’ pages	1 month (June 2015)	QL/QT	5
[49]	New Zealand	Facebook and YouTube	Children and adolescents (13–18 y)	1062 posts from 60 brands’ pages	24 months on YouTube (2015–2016) and 2 months on Facebook (October and November 2016)	QT	5
[50]	Thailand	Facebook	Children or/and adolescents	752 posts from 30 brands’ pages	1 month (December 2017)	QT	6
[51]	Australia	Facebook and Instagram	General	171 posts from 20 brands’ pages	~3 months-(19 February to 5 April 2018) and (25 June to 29 July 2018)	QT	6
[52]	Canada	Facebook	General	131 Facebook pages from 39 brands	12 months-June to August 2016	QT	4
[53]	United States	Facebook, Instagram, Twitter, Tumblr, and Vine	Children or/and adolescents	2000 posts from 20 brands’ pages	12 months (2016)	QL	7
[54]^1^	Spain	YouTube	Children or/and adolescents	304 videos from 13 brands’ pages and 15 YouTuber channels	1 Year (2019)	QL	3
[55]^1^	Spain	YouTube	Children or/and adolescents	304 videos from 13 brands’ pages and 15 YouTuber channels	1 Year (2019)	QL	3
[56]	New Zealand	Facebook, Instagram, YouTube and Twitter	General	372 posts from 20 brands’ pages	4 months (1 February to 31 May 2020)	QT	7
[57]	Canada	Facebook, Twitter, and Instagram	General	732 posts published on a family- friendly event page	2 months (January to February 2019)	QL	5
[58]	Uruguay	Facebook	General	619 posts from 96 brands’ pages	~3 months (14 March 2020 to 1 July 2020)	QL	7
[59]	Australia	Facebook, Instagram, YouTube, and Twitter	General	988 posts from 21 brands’ pages	4 months (February 1 and May 31, 2020)	QL	7
[60]	Unreported	Twitter	General	200 tweets from 10 brands’ pages	3 years (September 2016 and August 2019)	QL	7

Note: ^1^ Both publications use the same data sample; however, they assessed different advertising strategies. ^2^ This column presents the social media platforms studied by the selected publications; however, they may have assessed other digital media platforms such as websites, apps, and advergames. ^3^ The general category presented in this column is referred to studies that do not have a defined target audience or include more than one target audience. ^4^ Maximum of 7 stars. QT: Quantitative; QL: Qualitative.

**Table 2:** Brands, channels, or accounts, type of food and drinks evaluated.

Author	Food or Beverages Brands Information		Sample Definition Criteria
[35]	Energy-dense and nutrient-poor food and beverage brands	Subway, Red Bull, Monster Energy, Starburst, Nutella, Oreo, Maltesers, Bubble O’Bill Ice Creams, Cadbury Dairy Milk, Maltesers, Skittles, McDonald’s, Pringles, Domino’s Pizza, Vegemite, Slurpee, KFC, Subway, V Energy Drink, Coca-Cola, Hungry Jack’s, Slurpee, Pizza Hut, Ferrero Rocher, and Cold Rock Ice Creamery, Cadbury Eyebrows	The most liked pages on Facebook by Australians
[36]	Fast-food, confectionery, and soft drinks brands	McDonald’s, Cadbury Dairy Milk (Cadbury), and Coca-Cola	The three top selling brands in Australia
[37]	High fat, sugar, and salt food brands	Coca-Cola South Pacific Pty Ltd., McDonald’s Corporation, Cadbury Australia (Kraft Foods Australia Ltd.), Chupa Chups, Doritos Australia (The Smith’s Snackfood Company Ltd.), and Pringles (Kellogg Company)	(i) Food manufacturers listed among the top ten food advertisers in their categories; (ii) amount spent on advertising; (iii) frequency of advertising in other media and top-selling grocery brands
[38]	Soda brand	Coca-Cola	A prominent organization with divergent sustainability commitments.
[39]	Brand of a multinational chain of coffeehouses	Starbucks	A popular brand in the world, with a huge number of followers on Twitter
[40]	Fast-food brand	Kentucky Fried Chicken (KFC)	An example of quick-service restaurant, a sector at the forefront of online communications targeting the youth
[41]	Not mentioned	Not mentioned	The most liked pages on Facebook by Peruvians
[42]	Ultra-processed foods and beverages brands	Coca-Cola, Guaraná Antártica, Garoto, Cacau Show, Lacta official, McDonald’s, Bis, Halls, Subway, Trident, Guaraná Kuat oh yeah, Burger King, Kibon, Mundo Fini, Outback, and Mentos	The most liked pages on Facebook by Brazilians
[43]	Not mentioned	Not mentioned	All food and beverage advertisements presented on a university’s social media page
[44]	Not mentioned	Not mentioned	The most popular child-centric YouTube channels
[45]	Energy-dense, nutrient-poor food and beverage brands	Ben and Jerry’s, Burger King, Coca-Cola, Dominos, Gatorade, KFC, McDonald’s, Monster Energy, Nutella, Oreo, Pepsi, Red Bull, Starbucks, Subway, and Taco Bell	(i) Global sales rankings; (ii) presence of an account on Instagram; (iii) at least 100,000 followers; (iii) posts during the study period
[46]	Ten food industry brands Facebook and seven food industry brands Instagram	Not mentioned	The most liked pages on Facebook and Instagram by Australians
[47]	Sugar-sweetened beverage brands	Coca-Cola Australia, Pepsi Australia, Powerade Australia, Gatorade Australia, Red Bull, and Monster Energy	The most liked pages on Facebook by Australians
[48]	Energy drink brands	Not mentioned	The brands that were available in the two largest Australian retailers and service stations
[49]	Packaged food, fast-food, and beverage brands	*Facebook:* Whittaker’s Chocolate Lovers, Griff in’s, Cadbury Dairy Milk, Tip Top Ice Cream, Skittles, Chupa Chups, Lewis Road Creamery, Nutri-Grain, Kiwi Bacon, Pringles, Tasti, Puhoi Valley, Ferrero Rocher, KitKat, Marmite, McDonald’s, KFC, Domino’s, Pizza Hut, Subway, Burger King, Carl’s Jr., Pita Pit, BurgerFuel, Starbucks, Hell Pizza, Subway, Nando’s, Mexico, Wendy’s, Coca Cola, Red Bull, Lemon & Paeroa, V Energy, Monster Energy, Mountain Dew, Powerade, V Energy Drink, Pepsi, Charlie’s Drinks, Dr Pepper, Lipton Ice Tea, Phoenix Drinks, Gatorade, Sprite *YouTube:* KitKat, Tic Tac, Whittaker’s Chocolate, Weetbix, Anchor, Hell Pizza, Maccas, KFC, Carl’s Jr., Domino’s, V Energy, Coke Happiness, Nescafe, Sprite, Mountain Dew	The most liked pages on Facebook by New Zealanders and YouTube channels
[50]	Confectionery, soft drink, and retail food brands	Lays, Magnum, Cornetto, Glico, Wall’s, KitKat, Voiz, Twisties Cheetos, Glico ice TH, Nestle Ice Cream TH, Oishi Drink Station, ICHITAN, Coca-Cola, Pepsi, Big Cola, Est, Puriku, Sponsor, Fanta, 100PlusThailand, KFC, McDonald’s, Starbucks, SizzlerThai, The Pizza Company 1112 Lovers, Burger King, Pizza Hut, Dairy Queen, We Love Swensen’s, and Hot Pot Buffet	The most liked food and beverage pages on Facebook by Thai users
[51]	Not mentioned	Not mentioned	The most liked pages on Facebook by Australians
[52]	Packaged food, beverage, fast-food, restaurants, soft drink, and confectionary brands and brands with different products	Campbell Company, Coca-Cola, Danone, Ferrero, General Mills, Hersheys, Kellogg’s, Kraft, Maple Leaf Foods, Mars, McDonald’s, Mondelēz, Nestlé, Parmalat, Pepsico, Post Foods, Unilever Food Division, Weston Foods, Agropur Co-Op, Arla Foods Inc, A&W Food Services, Burger King, Cara Operations LTD, Con-Agra Foods, Dairy Farmers of Canada, Dairy Queen Restaurant, Dare Foods, Canada Dry Mott’s Inc. (Dr. Pepper Snapple Group), Fromageries Bel Sa, Hormel Foods, Mccain Foods, Quizno’s Corp, Subway, Tim Horton’s Canada, Wendy’s company (Triarc), Yum! Brands Inc, Darden Restaurants Inc., RedBull LTD., Storck International	(i) Companies participating in the Children’s Food & Beverage Advertising Initiative–CAI in Canada; (ii) CAI non-participating companies that were ranked as the top food and beverage advertisers on four children and youth television stations in Toronto
[53]	Fast-food, beverage, and snack brands	Coke Zero, Coca-Cola, Monster, Coke Life, Pepsi, Starbucks, Diet Coke, Fanta, Chick-fil-A, Dr Pepper, Red Bull, Mountain Dew, Dairy Queen, Sprite, Subway, Taco Bell, McDonald’s, KFC, Burger King, and Wendy’s	The food brands with the highest advertising expenditures in the United States
[54]^a^	Packaged food, sweets, and beverage brands and brands with different products	Nestlé, Casa Tarradellas, Grefusa, Nocilla, Dino Aventuras Danonino, The Phoskiters by Phoskitos, Nesquik, Kellogg’s, Cola-Cola, Hero, Adams Foods, Dulcesol, Galletas Gullón and more 15 Spanish channels Child YouTuber	The channels of food brands and child YouTubers with the most views and followers
[55]^a^	Not mentioned	Not mentioned	The channels of food brands and child YouTubers with the most views and followers
[56]	Confectionery, snacks, sugary drinks, and fast-food brands	Cadbury, Whittaker’s, Lindt, KitKat (Nestlé), M&Ms (Mars), Bluebird, Eta, Arnott’s, Doritos, Griffin’, Coca-Cola, Schweppes, Sprite, L&P, V (Frucor), McDonald’s, KFC, Subway, Burger King, Domino’s	Brands with the highest retail value in 2019
[57]	Not mentioned	Not mentioned	Advertisements related to Winterlude (family-friendly event)
[58]	Ultra-processed foods and beverages brands	Not mentioned	Ultra-processed products commercialised in the two largest and most popular online supermarkets
[59]	Confectionery, snacks, soft drinks, quick service restaurants, and food delivery services brands	Cadbury, Allen’s, Darrell Lea, Lindt, Mondelez, Nestlé, Unilever, Arnott’s, Coca-Cola, Pepsi Max, Coca-Cola Amatil Ltd., McDonald’s, KFC, Hungry Jack’s, Domino’s, Subway, Yum! Brands Inc, Uber Eats, Deliveroo, Menulog, DoorDash	Top five brands in sales in Australia and their parent companies
[60]	Packaged food and fast-food brands	Wendy’s, Burger King, DiGiorno, Oreo, Taco Bell, Denny’s, MoonPie, Hamburger Helper, Arby’s, Skittles	Search Google to identify media articles related to food or beverage brands

Note: **^a^** Both publications use the same data sample; however, they assessed different advertising strategies.

**Table 3:** Definitions of advertising strategies used by the publications included in the review.

Strategies	Definitions
**Brand in evidence**	
Branding elements	Presence of brands’ elements such as any characters featured on the page developed by the brand, logos, colours, trademarks, or slogans
Informational points	Presence of information about brands (official announcement, company news, and claims of awards won)
**Post Features**	
Audiovisual and graphics resources	Presence of resources such as photos, videos, texts, memes, and GIFs
Nature of post	Presence of elements that demonstrate the nature of the post (paid, or does not involve any sponsorship or is organic)
Tone of post	Presence of elements that qualify the tone of the post (positive, negative, neutral, optimistic, serious, and informal)
Presence of products, food, or beverages	Display of food or drinks in real content (photos) and graphics (emoticons)
Type of food	Classification of the types of food that were advertised, according to country’s dietary guidelines or food and beverage groups (vegetables, fruits, dairy, or protein-rich foods, grains, unhealthy food and beverage, beverage sugar, beverage low sugar, and sugar-free beverage)
Content topic	Post content, for example, content related to well-being and health (physical activity, body image, weight loss, and food), entertainment (music, humour, and pop culture), and informative (statistics or facts, sharing personal information or something relatable content creators’ life)
Presence of individuals	Presence of individuals, such as children, teenagers, young people, adults, and family, in the post
Post origin	Presence of elements that demonstrate whether the post was made by a user or brand, or if the brand shared content posted by the user (repost)
**Connectivity and engagement**	
Media tools	Presence of interactive media resources (applications, games, links, hashtag, and emoticons)
Incentive for an action (online)	Presence of incentive for users to take an online action such as request in the post to like, comment, or share content, request to respond to a poll, and incentive to perform a download
Conversations	Brand’s page administrator directly talks to users through comments
Challenges	Presence of content showing an action of the protagonist (YouTubers) who was challenged (by themselves or by another YouTuber) to perform an action (e.g., Child YouTubers who were challenged to eat foods of a single colour in one day)
**Economic advantages, gifts, competitions**	
Economic advantages, gifts, and competitions	Presence of resources such as limited-time offers, discount menus, 2-for-1 deals, or other reduced-price advertisements, advertisements to competitions, contests, premiums, samples, coupons, rebates and sweepstakes, and gifts and collectibles
**Promotional characters**	
Celebrities	Presence of people with an entertainment or media profile such as actors, singers, presenters
Sportspeople	Presence of amateur sportspersons or famous sportspersons or teams
Children’s characters	Presence of children’s characters from movies, books, television, and the Internet
Events	Presence of post announcing sports events, non-sporting events and festivals, holidays, or specific Facebook category in which page owners can create events and invite page members
Sponsorships and partnerships	Presence of any events that the brand supports or other brands or services the brand partners with, excluding charitable organizations
Cartoons or company owned characters or licensed characters	Presence of brand identification characters, licensed characters, and unlicensed characters
Movie	Presence of posts with mentions of movies
**Claims**	
Affective relationships	Presence of elements that associate the brand with the family relationship or encourages feelings of friendship
Encouragement to action	Presence of elements that encourage individuals to perform an action in different contexts, such as performing physical exercises or radical activities, starting a healthy diet, increasing water intake, and preparing a meal
Feelings and emotions	Presence of elements that associate the brand with feelings and emotions, such as enjoyment, happiness, superiority, acceptance by peers, friendships, motivation, pleasure, attraction, fear, indignation, serenity, and peace
Product characteristics	Presence of information about the product characteristics (i.e., looks, tastes, and ingredients), and statements about the brand, product, food composition, or health benefits (e.g., exclusive products, the best, the number one brand, new, associated with better health, improvements to the person’s diet or weight status, calories, sugar, fat, and carbohydrates)
**COVID-19**	
Hygiene	Presence of references to reducing chances of virus spread through hygiene practices when preparing food or handling food and drinks, social distancing by employees and customers (e.g., providing contactless or zero-contact services, keeping community safe), or references to the brand or company making hand sanitizer
Trading and events updates	Presence of references about practical updates around trading hours, the opening or closing of stores, and events during the COVID-19 pandemic (excluding delivery)
Encouraging messages	Presence of references of standing together during these challenging, unprecedented, or unexpected times, and the brand or company being there to support consumers, confirming that we are all in this together, or the inclusion of encouraging messages to raise positive emotions and associations in the context of the crisis generated by the COVID-19 pandemic
Care	Presence of textual or visual references to measures for the prevention of the transmission of COVID-19, or posts on health, including mental health advice with reference to COVID-19
Home delivery and take away	Presence of references to home delivery/take away in lockdown period/ no need to leave the house/using Uber eats (should include a specific mention of, for example, unusual times; difficult times/ staying at home, etc.)
Health care staff or essential workers	Thanking essential workers, health workers, and frontline workers, among others, and providing discounts for these workers
Corporate social responsibility	Presence of charitable work undertaken by the brands in the context of economic crisis generated by the COVID-19 pandemic (monetary donation or donating products)
Isolation activities	Presence of suggestions for things to do while in isolation/social distancing that relate to or include brand use or promotion: for example, recipes
Supporting local business and trading partners	Presence of references that suggest that consumers should support local businesses or announcements stating that the brand or company supports local businesses or their trading partners
Maintaining essential supply chain and commitment of the brand	Presence of references to the maintenance of supply chains, ensuring that consumers’ needs are met
**Corporate social responsibility (CSR) or philanthropy**	
Corporate social responsibility or philanthropy	Presence of references to charitable work undertaken by brand (direct donations to a charity or cause, and sales business revenue to promote a specific cause)

**Table 4:** Advertising strategies in general sample and stratified by social media and the target audience.

Advertising Strategies	General Sample (n = 24) ^1^	Social Media ^2^				Target Audience		
		Facebook (n = 7)	Twitter (n = 3)	Instagram or YouTube (n = 5)	Multiple (n = 9) ^3^	Children’s or/and Adolescents (n = 9)	Young Adults (n = 4)	General ^4^ (n = 11)
	**n**	**n**	**n**	**n**	**n**	**n**	**n**	**n**
**Brand in evidence**	**8**	**3**	**1**	**2**	**2**	**3**	**2**	**3**
Branding elements	5	3	0	1	1	1	2	2
Informational points	3	0	1	1	1	2	0	1
**Post Features**	**18**	**5**	**2**	**4**	**7**	**6**	**4**	**8**
Audiovisual and graphics resources	14	5	2	3	4	4	3	7
Nature of post	2	0	0	0	2	0	0	2
Tone of post	4	0	1	1	2	1	0	3
Presence of products, food, or beverages	8	1	0	2	5	3	2	3
Type of food	3	1	0	1	1	1	1	1
Content topic	5	0	1	1	3	1	1	3
Presence of individuals	6	1	0	1	4	2	0	4
Post origin	7	2	1	3	1	2	1	4
**Connectivity and engagement**	**18**	**6**	**2**	**3**	**7**	**6**	**4**	**8**
Media tools	11	4	0	2	5	5	2	4
Incentive for an action (online)	8	3	1	0	4	2	1	5
Conversations	8	4	1	1	2	1	2	5
Challenges	1	0	0	1	0	1	0	0
**Economic advantages, gifts, and competitions**	**14**	**6**	**1**	**3**	**4**	**6**	**2**	**6**
Economic advantages, gifts, and competitions	14	6	1	3	4	6	2	6
**Promotional characters**	**12**	**4**	**1**	**2**	**5**	**4**	**2**	**6**
Celebrities	8	3	0	2	3	4	1	3
Sportspeople	7	3	0	2	2	3	2	2
Children’s characters	4	2	0	1	1	0	1	3
Events	7	3	1	0	3	2	0	5
Sponsorships and partnerships	4	3	0	1	0	1	1	2
Cartoons or company owned characters or licensed characters	3	0	0	1	2	2	1	0
Movie	1	0	0	0	1	1	0	0
**Claims**	**14**	**2**	**2**	**4**	**6**	**6**	**3**	**5**
Affective relationships	4	1	0	2	1	3	0	1
Encouragement to action	5	1	0	1	3	3	0	2
Feelings and emotions	10	2	2	3	3	4	2	4
Product characteristics	8	1	1	4	2	5	1	2
**COVID-19**	**3**	**1**	**0**	**0**	**2**	**0**	**0**	**3**
Hygiene	2	0	0	0	2	0	0	2
Trading and events updates	2	0	0	0	2	0	0	2
Encouraging messages	3	1	0	0	2	0	0	3
Care	2	1	0	0	1	0	0	2
Home delivery and take away	3	1	0	0	2	0	0	3
Health care staff or essential workers	2	0	0	0	2	0	0	2
Corporate social responsibility	3	1	0	0	2	0	0	3
Isolation activities	3	1	0	0	2	0	0	3
Supporting local business and trading partners	2	0	0	0	2	0	0	2
Maintaining essential supply chain and commitment of the brand	3	1	0	0	2	0	0	3
**Corporate social responsibility (CSR) or philanthropy**	**7**	**3**	**1**	**2**	**1**	**3**	**1**	**3**
Corporate social responsibility or philanthropy	7	3	1	2	1	3	1	3

Notes: ^1^ Two studies [34,50] were not included in this table because it was not possible to identify the advertising strategies stratified by social media; ^2^ grouped according to media profile based on Zuh and Chen (2015) [31]; ^3^ when a study evaluated more than one social media; ^4^ the general category presented in this column is referred to studies that do not have a defined target audience or include more than one target audience.

## Data Availability

No new data were created or analyzed in this study. Data sharing is not applicable to this article.

## Supplementary Materials

The following supporting information can be downloaded at: https://www.mdpi.com/article/10.3390/ijerph20043615/s1, Supplementary material S1: Search strategies used on each database; Supplementary material S2: Newcastle-Ottawa Scale adapted for food advertising monitoring studies on social media; Supplementary material S3: Assessment of risk for bias.
