# Supplementary material for: A Scoping Review of Observational Studies on Food and Beverage Advertising on Social Media: A Public Health Perspective
Source: Int J Environ Res Public Health. 2023 Feb 17;20(4):3615. doi: 10.3390/ijerph20043615 (PMC9960697; doi:10.3390/ijerph20043615)
Supplement: Supplementary file 1 [file ijerph-20-03615-s001.zip › Supplementary material S1.pdf]

Supplementary material S1.

Search strategies used on each database

"Social Media"[Mesh] OR "Social Media" OR "Media, Social" OR "Social Medium" OR "Twitter Messaging" OR "Messaging, Twitter" OR "Web 2.0" OR "2.0s, Web" OR "Web 2.0s" OR “Online” OR “Facebook” OR “Instagram” OR “Twitter” OR “Youtube” AND "Food"[Mesh] OR "Food" OR "Foods" OR "Fast Foods"[Mesh] OR "Fast Foods" OR "Fast Food" OR (Food, Fast) OR (Foods, Fast) OR "Convenience Foods" OR "Convenience Food" OR (Food, Convenience) OR (Foods, Convenience) OR (Ready-Prepared Foods) OR (Food, Ready-Prepared) OR (Foods, Ready-Prepared) OR "Ready Prepared Foods" OR "Ready-Prepared Food" OR "Ready-To-Eat Meals" OR "Ready To Eat Meals" OR (Meals, Ready-To-Eat) OR (Meal, Ready-To-Eat) OR (Meals, Ready To Eat) OR "Ready-To-Eat Meal" OR "Food and Beverages"[Mesh] OR "Food and Beverages" Or "Beverages and Food" OR "Beverages"[Mesh] Or "Beverages" OR "Beverage" OR "Carbonated Beverages"[Mesh] OR "Carbonated Beverages" OR (Beverage, Carbonated) OR (Beverages, Carbonated) OR "Carbonated Beverage" OR "Carbonated Drinks" OR "Carbonated Drink" OR (Drink, Carbonated) OR "Drinks, Carbonated" OR "Soft Drinks" OR "Drink, Soft" OR "Drinks, Soft" OR "Soft Drink" OR "Soda Pop" OR "Pop, Soda" OR (Pops, Soda) OR "Soda Pops" OR "Artificially Sweetened Beverages"[Mesh] OR "Artificially Sweetened Beverages" OR "Artificially Sweetened Beverage" OR (Beverage, Artificially Sweetened) OR (Beverages, Artificially Sweetened) OR (Sweetened Beverage, Artificially) OR (Sweetened Beverages, Artificially) OR "Diet Drinks" OR "Diet Drink" OR (Drink, Diet) OR "Drinks, Diet" OR "Diet Beverages" OR "Beverage, Diet" OR "Beverages, Diet" OR "Diet Beverage" OR "Sugar-Sweetened Beverages"[Mesh] OR "Sugar-Sweetened Beverages" OR (Beverage, Sugar-Sweetened) OR (Beverages, Sugar-Sweetened) OR "Sugar Sweetened Beverages" OR "Sugar-Sweetened Beverage" OR "Sugar-Added Beverages" OR (Beverage, Sugar-Added) OR (Beverages, Sugar-Added) OR "Sugar Added Beverages" OR (Sugar-Added Beverage) OR "Sweetened Drinks" OR (Drink, Sweetened) OR (Drinks, Sweetened) OR "Sweetened Drink" OR "Sugar-Sweetened Soft Drinks" OR (Drink, Sugar-Sweetened Soft) OR (Drinks, Sugar-Sweetened Soft) OR "Soft Drink, Sugar-Sweetened" OR "Soft Drinks, Sugar-Sweetened" OR "Sugar Sweetened Soft Drinks" OR "Sugar-Sweetened Soft Drink" OR "Sweetened Beverages" OR (Beverage, Sweetened) OR (Beverages, Sweetened) OR "Sweetened Beverage" OR "Sugar-Sweetened Sodas" OR (Soda, Sugar-Sweetened) OR (Sodas, Sugar-Sweetened) OR "Sugar Sweetened Sodas" OR "Sugar-Sweetened Soda" OR "Food Industry"[Mesh] OR "Food Industry" OR "Food Industries" OR "Industries, Food" OR "Industry, Food" OR "Food-Processing Industry"[Mesh] OR "Food-Processing Industry" OR "Food Processing Industry" OR (Industry, Food-Processing) OR "Food-Processing Industries" OR (Industries, Food-Processing) OR (Industry, Food Processing) OR “Meals”[Mesh] OR “Meals” OR “Meal” OR “Mealtimes” OR “Mealtimes” OR “Meal Times” OR “Time, Meal” OR (Times, Meal) OR “Meal Time” OR “Mealtime” OR “Dinner Time” OR “Dinner Times” OR “Time, Dinner” OR (Times, Dinner) OR “Dinnertime” OR “Dinnertimes” OR “Dinner” OR “Dinners” OR “Supper” OR “Suppers” OR "Snacks"[Mesh] OR "Snacks" OR “Snack” OR “Snack Food” OR “Food, Snack” OR “Foods, Snack” OR “Snack Foods” OR “Snacktime” OR “Snacking” OR “Food brands” OR “Food companies” OR “Coca-Cola” OR “PepsiCo” OR “Mondelez” OR “Unilever” OR “Nestlé” OR “Danone” OR “Chips” OR “McDonald’s” OR “Burger King” OR “Subway” OR “Kentucky Fried Chicken” OR “Starbucks” OR “Ultra-processed food” OR “Industrialized foods” AND "Marketing"[Mesh] OR "Marketing" OR "Market Research" OR "Research, Market" OR "Audience Research" OR (Research, Audience) OR "Advertising”[Mesh] OR "Advertising" OR "Advertisings" OR "Telemarketing" OR “Propaganda"[Mesh]

OR “Propaganda" OR “Advertisement” OR "Advertisements" OR "Communication”[Mesh] OR "Personal Communication" OR (Communication, Personal) OR "Misinformation" OR "Communication Programs" OR "Communication Program" OR (Program, Communication) OR "Programs, Communication" OR "Communications Personnel" OR (Personnel, Communications) OR “Persuasive communication”[Mesh] OR “Persuasive communication” OR (Communication, persuasive) OR “Persuasion” OR “Brainwashing” OR "Information Dissemination"[Mesh] OR "Information Dissemination" OR "Dissemination, Information" OR "Information Distribution" OR "Distribution, Information" OR "Information Sharing" OR "Information Sharing" OR "Sharing, Information" OR (Sharings, Information) OR "Data Sharing" OR "Data Sharing" OR (Sharing, Data) OR (Sharings, Data) OR “Digital marketing” OR “Digital advertising” OR “Digital media” OR “Marketing techniques” OR “Marketing strategies” OR “Marketing techniques” OR “Marketing strategies” OR “Trend Marketing” OR “Vehicles of marketing” OR “Publicity” OR “Persuasive marketing techniques” OR "Advertisement" [Publication Type] OR “Advertisement” OR “Food marketing” OR “Junk Food Marketing” OR “Food advertising” OR “Sports Sponsorship food” OR “Sports Sponsorship” OR “Food Sponsorships” OR “Fast food marketing” OR “Unhealthy food marketing” OR “Unhealthy food and beverages marketing” OR “Food and beverages marketing” OR “Junk food marketing” OR “Food advertisements” OR “Marketing of food” OR “Influencer Marketing of Food” OR “Food Publicity” OR “Food promotion” OR “Persuasive food marketing” OR “Monitoring food marketing” OR “Power food marketing” OR “Online food communication”
