# Supplementary material for: A Scoping Review of Observational Studies on Food and Beverage Advertising on Social Media: A Public Health Perspective
Source: Int J Environ Res Public Health. 2023 Feb 17;20(4):3615. doi: 10.3390/ijerph20043615 (PMC9960697; doi:10.3390/ijerph20043615)
Supplement: Supplementary file 1 [file ijerph-20-03615-s001.zip › Supplementary material S2.pdf]

Supplementary material S2. Newcastle-Ottawa Scale adapted for food advertising monitoring studies on social media

I Selection: (Maximum 5 stars)

1. Sample representativeness (assesses the representativeness of the sample according to the aim of the study and the number of media and brands studied):

a) Representative (the number of media and brands studied is adequate to achieve the objective of the study - e.g. study aimed to explore the nature of appealing strategies used by the food industry on digital platforms. In this case, the study needs to explore more than one social media and food brand). *

b) Selected group (the number of media and brands studied is limited according to accomplish the aim of the study – e.g. in the same case mentioned above, the authors chose only one media or brand among several other possibilities. The choices were not justified).

2. Sample analysis period (data collection was assessed in an acceptable length of time according to the objective of the study):

a) A year or more of data collection (e.g. study aimed to explore the nature of appealing strategies used by the food industry on digital platforms independently of special days, holidays and others). *

b) Less than a year of data collection and length was justified (e.g. study aimed to describe food advertising during a sporting event)*.

c) Less than a year of data collection and length was not justified.

3. Identification of the exposure (refers to the tools used to identify the strategy):

a) Validated measurement tool – qualitative or quantitative (i.e. if one or more of the following criteria are met: (i) content analysis based on literature; (ii) a pilot was run; (iii) based on previous studies). **

b) No description of the measurement tool.

4. Definition of the exposure (refers to the detailed definition of the strategy studied):

a) Definition of the advertising strategies (i.e. if one or more of the following criteria are met: (i) a table with a definition of each strategy; (ii) detailed examples of the strategies, (iii) supplementary material describing the advertising strategies). *

b) No definition of the advertising strategies.

II Outcome: (Maximum 2 stars)

1. Assessment of the outcome (refers the methodology used by the research team to analyze data on advertising strategies):

a) Independent blind assessment (full analyses were conducted by two or more researchers, with the results compared and the divergences verified) or Concordance Analysis in a part of the sample. **

b) Training conducted before data coding. *

c) No description.

This scale has been adapted from the Newcastle-Ottawa Quality Assessment Scale for cohort studies (Wells et al.) to perform a quality assessment of food advertising monitoring studies on social media for the systematic review “A scoping review of observational studies on food and beverage advertising on social media”.
