# Supplementary material for: A Scoping Review of Observational Studies on Food and Beverage Advertising on Social Media: A Public Health Perspective
Source: Int J Environ Res Public Health. 2023 Feb 17;20(4):3615. doi: 10.3390/ijerph20043615 (PMC9960697; doi:10.3390/ijerph20043615)
Supplement: Supplementary file 1 [file ijerph-20-03615-s001.zip › Supplementary material S3.pdf]

Supplementary material S3. Assessment of risk for bias.

Author (year)	Selection: (Maximum 5 stars)				Outcome: (Maximum 2 stars)	Total Score (Maximum 7 stars)
	Sample representativeness	Sample analysis period	Identification of the exposure	Definition of the exposure	Assessment of the outcome	
Freeman et al. (2014)	*	*	**	*		5
Boelsen-Robinson et al. (2016)	*		**	*	**	6
Confos and Davis (2016)	*		**	*	**	6
Austin and Gaither (2016)	*	*	**	*	**	7
Taecharungroj (2017)	*	*	**	*	**	7
Laestadius and Wahl (2017)	*		**		**	5
Busse (2018)	*		**	*	**	6
Horta et al. (2018)	*	*	**	*	**	7
Jayanetti et al. (2018)	*	*	**			4
Tan et al. (2018)	*		**	*	**	6
Vassallo et al. (2018)	*	*	**	*	**	7
Klassen et al. (2018)	*		**	*	**	6
Brownbill et al. (2018)	*		**	*		4
Buchanan et al. (2018)	*		**		**	5
Vandevijvere et al. (2018)	*	*		*	**	5
Jaichuen et al. (2019)	*		**	*	**	6
Barklamb et al. (2020)	*		**	*	**	6
Kent et al. (2020)	*			*	**	4
Bragg et al. (2020)	*	*	**	*	**	7
Castelló-Martínez and Tur-Viñes. (2020a)	*	*		*		3
Gerritsen et al. (2021)	*	*	**	*	**	7
Amson et al. (2021)	*	*		*	**	5
Antunéz et al. (2021)	*	*	**	*	**	7
Martino et al. (2021)	*	*	**	*	**	7
Greene et al. (2021)	*	*	**	*	**	7

Castelló-Martínez and Tur-Viñes. (2021a)	*	*	*	3
